# Technical challenges regarding the use of formalin-fixed paraffin embedded (FFPE) tissue specimens for the detection of bacterial alterations in colorectal cancer

**DOI:** 10.1186/s12866-021-02359-z

**Published:** 2021-10-29

**Authors:** Suk Yee Lam, Athanasia Ioannou, Prokopis Konstanti, Thijmen Visseren, Michail Doukas, Maikel Petrus Peppelenbosch, Clara Belzer, Gwenny Manel Fuhler

**Affiliations:** 1 Department of Gastroenterology and Hepatology, Erasmus MC University Medical Center Rotterdam, P.O. Box 2040, 3000 CA Rotterdam, The Netherlands; 2 Laboratory of Microbiology, Wageningen University & Research, P.O. Box 8033, 6700 EH Wageningen, The Netherlands; 3 Department of Pathology, Erasmus MC University Medical Center Rotterdam, P.O. Box 2040, 3000 CA Rotterdam, The Netherlands

**Keywords:** DNA contamination, Gastrointestinal microbiome, High-throughput nucleotide sequencing, Formalin-fixed paraffin embedded, Low biomass, Colorectal neoplasms

## Abstract

**Background:**

Formalin-fixed paraffin embedded (FFPE) tissues may provide an exciting resource to study microbial associations in human disease, but the use of these low biomass specimens remains challenging. We aimed to reduce unintentional bacterial interference in molecular analysis of FFPE tissues and investigated the feasibility of conducting quantitative polymerase chain reaction (qPCR) and 16S rRNA amplicon sequencing using 14 colorectal cancer, 14 normal adjacent and 13 healthy control tissues.

**Results:**

Bacterial contaminants from the laboratory environment and the co-extraction of human DNA can affect bacterial analysis. The application of undiluted template improves bacterial DNA amplification, allowing the detection of specific bacterial markers (*Escherichia coli* and *Faecalibacterium prausnitzii*) by qPCR. Nested and non-nested PCR-based 16S rRNA amplicon sequencing approaches were employed, showing that bacterial communities of tissues and paired paraffin controls cluster separately at genus level on weighted Unifrac in both non-nested (R2 = 0.045; Pr(> F) = 0.053) and nested (R2 = 0.299; Pr(> F) = 0.001) PCR datasets. Nevertheless, considerable overlap of bacterial genera within tissues was seen with paraffin, DNA extraction negatives (non-nested PCR) or PCR negatives (nested PCR). Following mathematical decontamination, no differences in α- and β diversity were found between tumor, normal adjacent and control tissues.

**Conclusions:**

Bacterial marker analysis by qPCR seems feasible using non-normalized template, but 16S rRNA amplicon sequencing remains challenging. Critical evaluation of laboratory procedures and incorporation of positive and negative controls for bacterial analysis of FFPE tissues are essential for quality control and to account for bacterial contaminants.

**Supplementary Information:**

The online version contains supplementary material available at 10.1186/s12866-021-02359-z.

## Background

The preservation of formalin-fixed paraffin embedded (FFPE) tissue samples in the archives of health institutes has facilitated the study of human disease worldwide. In contrast to prospectively collected fresh and frozen material, FFPE tissue specimens are readily available to investigate a variety of health-related issues [1, 2]. Pathology archives are also an exciting potential source of information to answer microbe-related health questions. Both bacterial and viral deoxyribonucleic acid (DNA) can be detected in FFPE tissue specimens and have been used to investigate associations between invading pathogens and diseases, for instance to determine the presence of *Helicobacter pylori* in gastric adenocarcinoma [3] as well as hepatocellular carcinoma [4] and human papilloma virus in cervical cancer [5]. Since innovative technologies have enabled the identification of microbiota and their genomes (microbiome) in the different niches of the human body [6, 7], FFPE tissue specimens might serve as an additional source to map these communities. Research questions which require the investigation of specific disease sites, rare diseases or a long follow-up time of patients may in particular benefit from the use of long-term collection and storage of FFPE tissue material. Examples of such studies include the investigation of colorectal cancer (CRC)-specific microbial composition [8] and the exploration of intestinal bacterial communities in neonates with necrotising enterocolitis [9–11].

Nevertheless, the application of FFPE tissues for microbiome analyses is associated with several challenges. First, obtaining sufficient quantities of genomic DNA of good quality remains difficult [12–15]. Neutrally buffered formalin prevents total DNA degradation [2, 14], but DNA cross-linking and fragmentation [14, 16] as well as storage time post-fixation [15, 17] impair the recovery of nucleic acids. Whereas the amplification of large DNA fragments is considered problematic due to DNA integrity deterioration [12, 14], shorter fragments have been used for molecular analyses [15–19], even in samples archived for over twenty years [20]. Secondly, FFPE tissues have relatively high human genomic DNA content and are considered low bacterial biomass samples. Amplification steps such as nested polymerase chain reaction (PCR) may improve specificity and sensitivity of detected bacteria. Thirdly, microbial contaminants were shown to be present in commonly used reagents and can critically influence microbiome results, especially in low bacterial biomass samples [21–26]. Since bacterial contamination affects both 16S ribosomal RNA (rRNA) amplicon sequencing and shotgun metagenomics [25], careful handling of samples is essential during bacterial DNA retrieval and subsequent molecular analysis.

In this study, our aim is to optimise a method to reduce the interference of non-informative microbial contaminants in order to extract biologically relevant information from FFPE tissue specimens. To investigate the feasibility of conducting microbial analyses, we employed a cohort of 41 FFPE specimens to explore microbial associations in CRC using bacterial marker analysis and 16S rRNA amplicon sequencing. We report the difficulties encountered in an effort to optimize the processing of FFPE tissue specimens for future microbial studies.

## Results

### Bacterial and human DNA interference in microbial analyses of FFPE tissue specimens

**Fig. 1:**
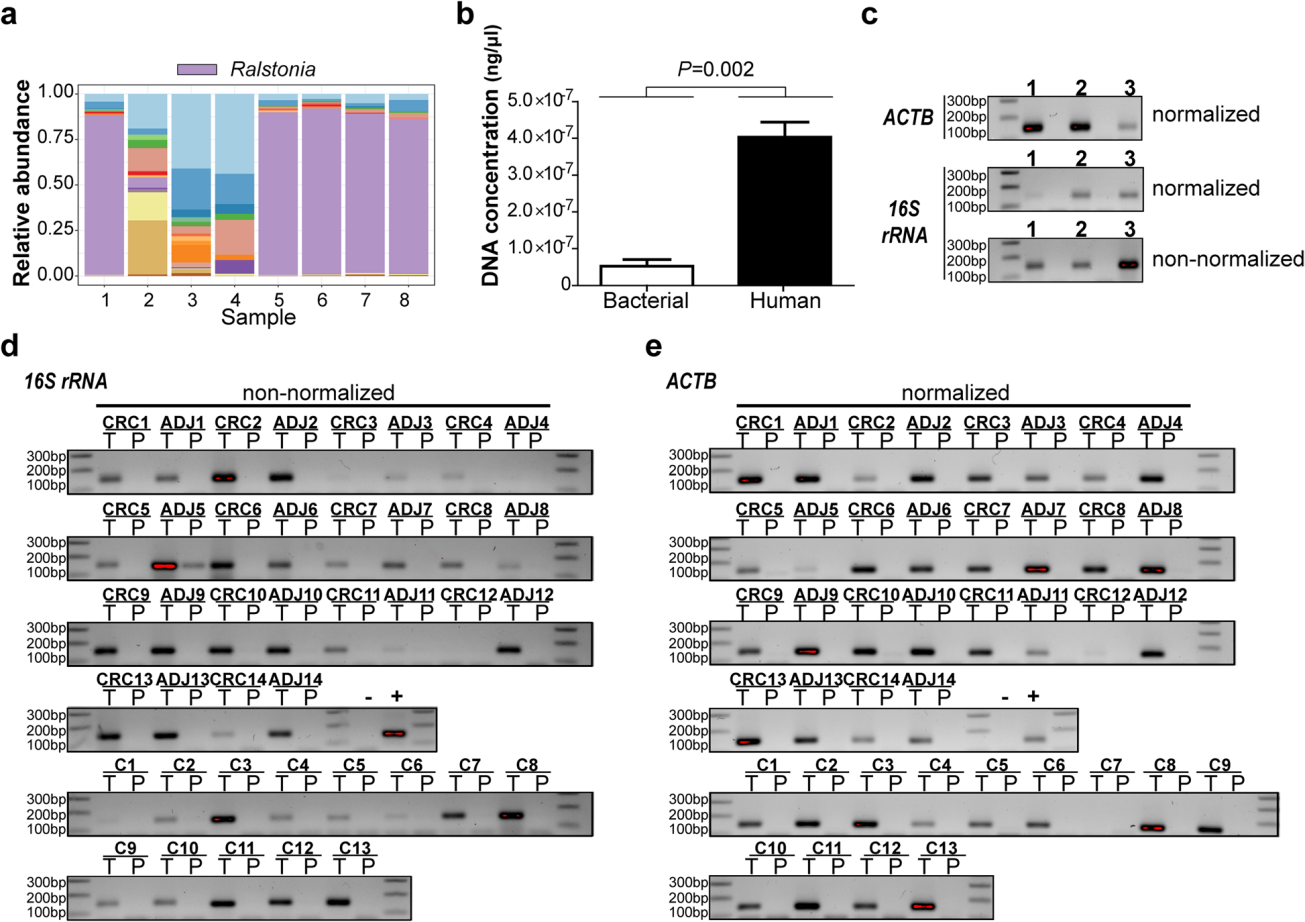
Unintentional bacterial and human DNA interference in molecular analyses of formalin-fixed paraffin embedded (FFPE) tissues. **a** 16S rRNA amplicon sequencing pilot results showing the relative abundance of the top 20 genera in FFPE tissue samples (n = 8). A predominance of genus *Ralstonia* is observed in a majority of samples. **b** Quantitative polymerase chain reaction (qPCR) results demonstrating the amount of bacterial versus human genomic DNA in normalized template (n = 3). Statistical significance was calculated using the unpaired t-test. **(c)** Gel electrophoresis results following *actin beta (ACTB)* and 16S rRNA gene amplification, emphasizing the use of non-normalized (undiluted) template to improve bacterial DNA detection in FFPE tissue samples (n = 3). **d-e** Gel electrophoresis results showing bacterial and human genomic DNA presence in 41 paired FFPE tissue (T) samples and their paired empty paraffin (P) controls. Tissues consist of colorectal cancer (CRC; n = 14), normal adjacent (ADJ; n = 14) and healthy control (C; n = 13) tissues. Full length gel electrophoresis results are shown in Additional Fig. A1

Interference from contaminants is a common problem for samples of low microbial biomass. The 16S rRNA amplicon sequencing pilot results showed a predominance of *Ralstonia* in five out of eight samples (Fig. 1a). Retrospective analysis demonstrated the elution buffer as the contaminating source while other extraction reagents were excluded (Supplementary Fig. S1a). Differences in bacterial DNA detection were observed for the same set of pathology paraffin collections using two separate kits (Supplementary Fig. S1b), indicating that the extent of contamination varies per newly opened kit and its components (Supplementary Fig. S1c), while paraffin itself is not a contaminating source. Nevertheless, bacterial DNA presence was confirmed in the majority of 41 FFPE tissues and was minor in paired paraffin controls (Fig. 1d), with quantification of the bacterial biomass showing significantly higher 16S rRNA gene copies numbers in tissues (n = 39) compared to matched paraffin samples (n = 38) (Supplementary Fig. S1d). However, human genomic DNA is co-extracted with bacterial DNA and present in higher concentrations in FFPE tissues (*P* = 0.002) (Fig. 1b, e), and therefore normalisation of samples for DNA concentration may hamper bacterial DNA detection (Fig. 1c). Thus, human and microbial contaminants may interfere in microbial analysis of low biomass samples and should be accounted for.

### Bacterial marker analysis of FFPE tissue specimens by qPCR

**Fig. 2:**
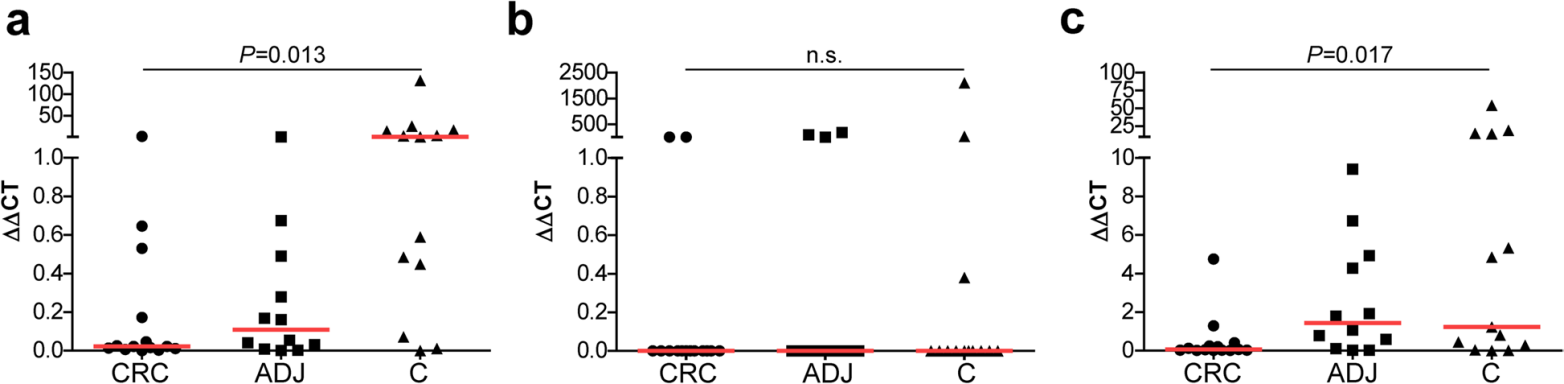
Quantitative polymerase chain reaction (qPCR) analysis of bacterial markers in formalin-fixed paraffin embedded (FFPE) tissues. qPCR results of specific bacterial markers in colorectal cancer (CRC; n = 14), normal adjacent (ADJ; n = 12) and healthy colonic control (C; n = 13) using non-normalized template. The relative amount of each sample is normalized to human genomic DNA and expressed in terms of fold change (2^−ΔΔCT^). **a**-**b** Detection of *Escherichia coli (E.coli)* (**a**) and strains harbouring the *pks +* island (**b**) using *E. coli* and *ClbA* gene specific primers, respectively. **c** Detection of *Faecalibacterium prausnitzii*

The use of non-normalized template allowed the comparison of bacterial markers in CRC (n = 14), normal adjacent tissues (n = 12) and healthy tissues (n = 13). The fold change (2^−ΔΔCT^) levels of *Escherichia coli (E.coli)* were significantly different among groups (*P* = 0.013), in particular CRC compared to healthy controls (Fig. 2a). A minority of tissue samples harboured *pks* positive strains, but no differences were detected among groups (Fig. 2b). The levels of *Faecalibacterium prausnitzii (F. prausnitzii)* differed between tissue types (*P* = 0.017) with post-hoc analysis indicating significant higher levels in healthy controls compared to CRC (Fig. 2c).

### Exploring bacterial communities in FFPE tissues, paraffin and controls with two sequencing approaches

**Fig. 3:**
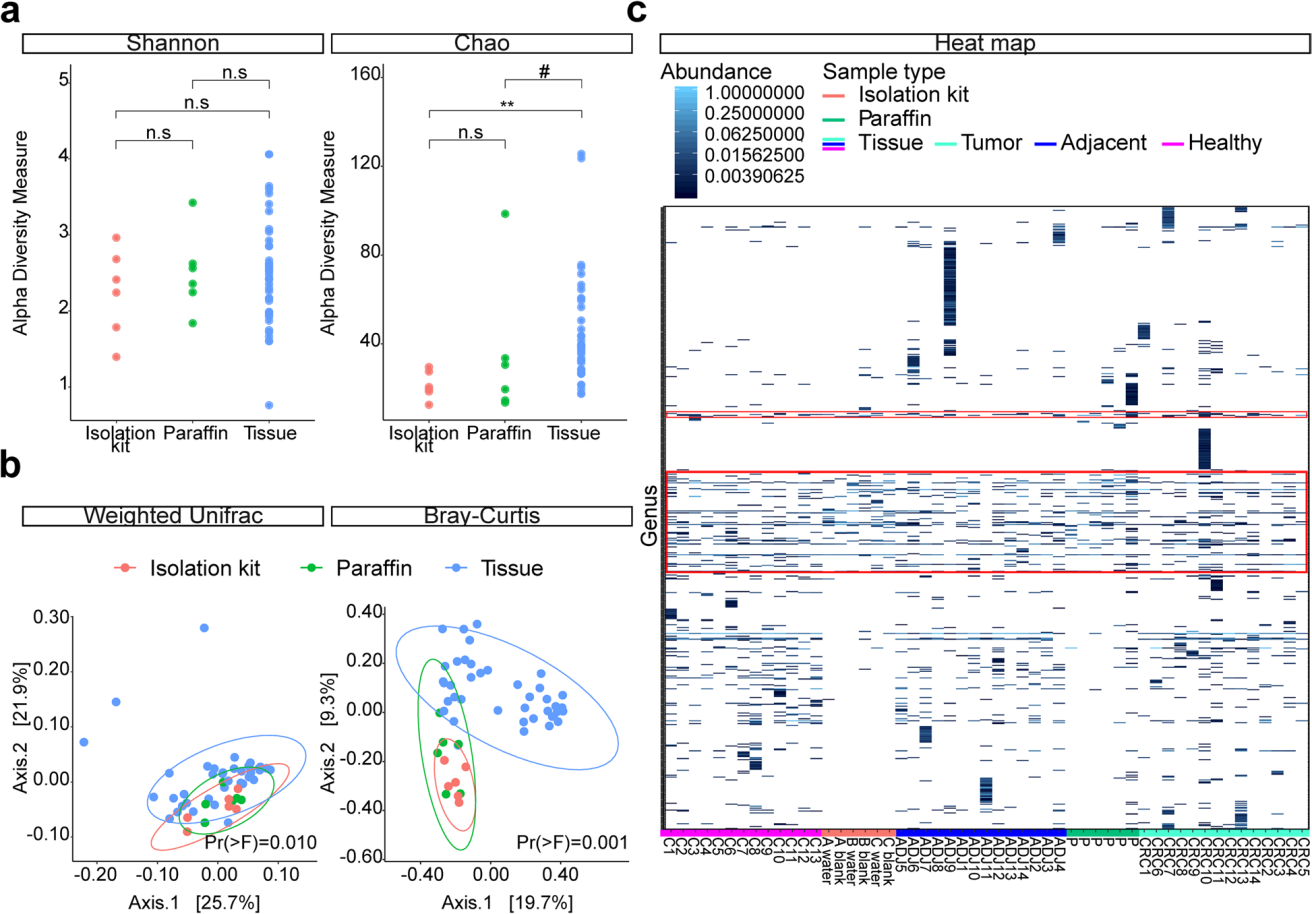
Targeted 16S rRNA gene amplification and sequencing approach for tissue, paraffin and DNA extraction negatives. The 16S rRNA amplicon sequencing results of formalin-fixed paraffin embedded (FFPE) tissue (n = 41), empty paraffin controls (n = 6) and DNA extraction controls (n = 6) at genus level using a non-nested polymerase chain reaction (PCR) approach. **a** Dot plots show the bacterial biodiversity measured by Shannon and Chao1 diversity indices. **b** Principal Coordinates Analysis (PCoA) plots illustrate the bacterial composition of tissue, paraffin and DNA extraction controls using weighted UniFrac and Bray-Curtis dissimilarity. **c** Heat map of the relative abundance of genera across samples. Tissue specimens consist of tumor (n = 14), normal adjacent tissue (n = 14) and healthy colonic tissue (n = 13). The red box indicates genera present in DNA extraction negatives and other samples. Abbreviations for level of significance: n.s. not significant; #, *P <* 0.1; **, *P <* 0.01

First, we employed the most commonly used sequencing approach, with amplification of the bacterial 16S rRNA hypervariable region V3-V4. A total of 41 FFPE tissue samples, six paraffin controls and six DNA extraction negatives were included for sequencing. No differences in Shannon diversity were found at genus level, but the Chao1 diversity index was significantly higher in tissues than DNA extraction negatives (*P* < 0.010) (Fig. 3a). The bacterial communities clustered separately based on weighted UniFrac (R2 = 0.088; Pr(> F) = 0.010) and Bray-Curtis dissimilarity (R2 = 0.116; Pr(> F) = 0.001), as shown by the Principal Coordinates Analysis (PCoA) plots at genus level (Fig. 3b). Pairwise comparisons indicated that tissues were distinct from DNA extraction negatives on weighted UniFrac (R2 = 0.063; Pr(> F) = 0.017) and Bray-Curtis dissimilarity (R2 = 0.081; Pr(> F) = 0.001). Tissues also differed from paraffin when computed with Bray-Curtis dissimilarity (R2 = 0.066; Pr(> F) = 0.001) while a trend was observed for weighted Unifrac (R2 = 0.045; Pr(> F) = 0.053). Homogeneity conditions were met and findings were in agreement at operational taxonomic unit (OTU) level (Supplementary Table S3). The heat map demonstrated a considerable overlap of genera among tissues, paraffin and DNA extraction negatives (Fig. 3c). A total of 53 bacterial families were detected in these latter controls, indicating significant interference from contaminants/artefacts derived during DNA isolation, library preparation and sequencing procedures (Supplementary Fig. S2a).

**Fig. 4:**
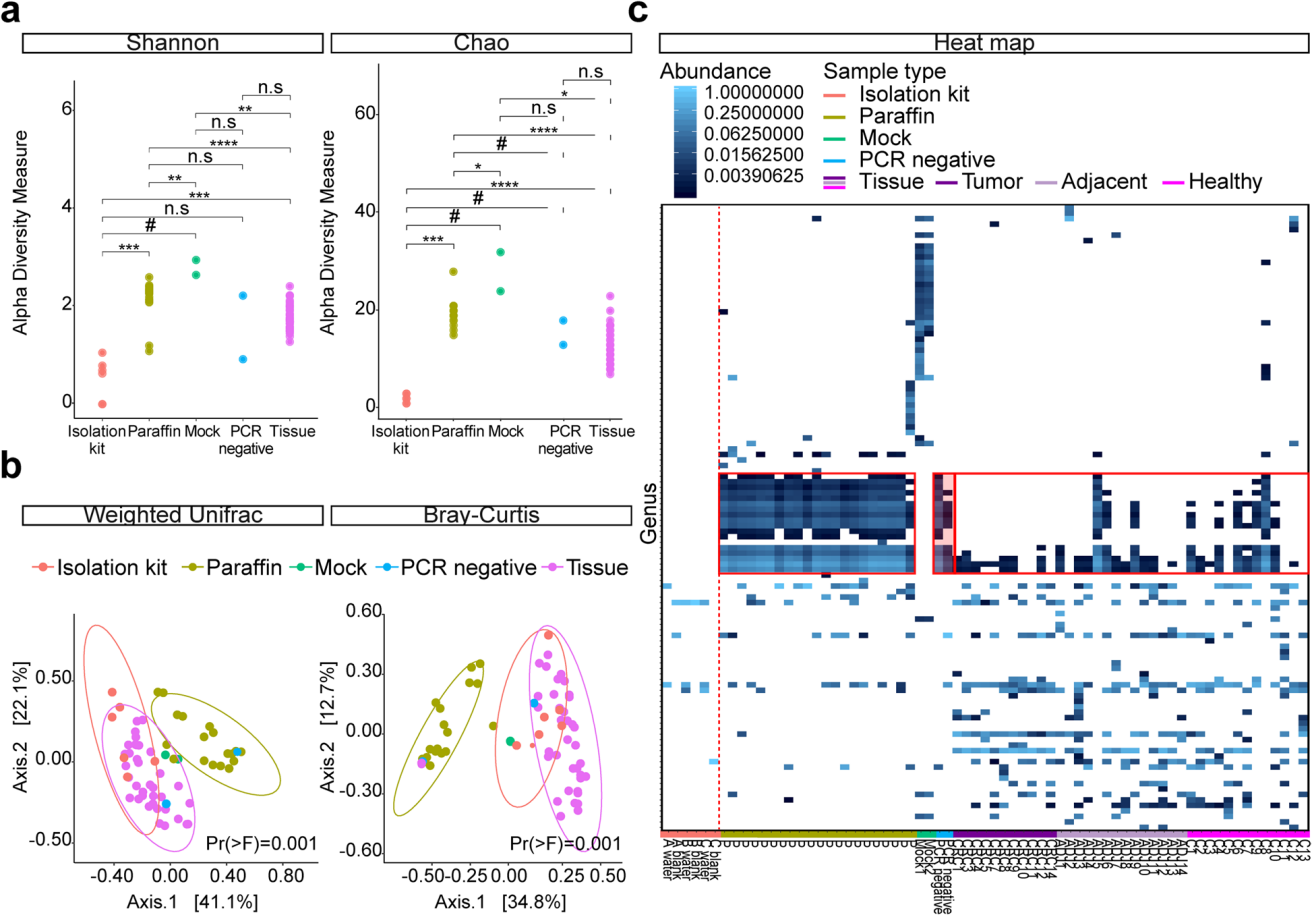
Nested 16S rRNA gene amplification and sequencing approach for tissue, paraffin and controls. The 16S rRNA amplicon sequencing results of formalin-fixed paraffin embedded (FFPE) tissue (*n* = 38), empty paraffin controls (*n* = 21), DNA extraction controls (*n* = 6), polymerase chain reaction (PCR) negatives (*n* = 2) and synthetic mock communities (*n* = 2) at genus level using a nested PCR approach. **a** Dot plots show the bacterial biodiversity measured by Shannon and Chao1 diversity indices. **b** Principal Coordinates Analysis (PCoA) plots illustrate the bacterial composition of tissue, paraffin and the different controls using weighted UniFrac and Bray-Curtis dissimilarity. **c** Heat map analysis of the relative abundance of genera across samples. Tissue specimens consist of tumor (*n* = 11), normal adjacent tissue (*n* = 14) and healthy colonic tissue (*n* = 13). The red box indicates the genera present in PCR negatives and other samples. The red dotted line separates the DNA extraction negatives that were processed separately during library preparation. Abbreviations for level of significance: n.s. not significant; #, *P <* 0.1; *, *P <* 0.05; **, *P <* 0.01; ***, *P* < 0.001; ****, *P* < 0.0001

A second approach using nested PCR for bacterial DNA amplification was performed on 38 FFPE tissues, 21 paraffin controls, two mock communities, six DNA extraction negatives (samples undergoing DNA isolation procedure) and two PCR negatives (samples not undergoing DNA isolation, i.e. PCR/sequencing controls). Shannon and Chao1 diversity indices were higher in tissues than DNA extraction negatives (*P* < 0.001 and *P* < 0.0001), but lower compared to paraffin (both *P* < 0.0001) (Fig. 4a). No differences in α-diversity were observed between tissues and PCR negatives. Moreover, the community structure among the five groups were significantly different based on weighted UniFrac (R2 = 0.374; Pr(> F) = 0.001) (Fig. 4b). Tissues were distinct from paraffin (R2 = 0.299; Pr(> F) = 0.001) and DNA extraction negatives (R2 = 0.155; Pr(> F) = 0.001), but not from PCR negatives (R2 = 0.055; Pr(> F) = 0.056). Also paraffin did not differ from these PCR controls (R2 = 0.061; Pr(> F) = 0.234). When using Bray-Curtis dissimilarity for overall group comparison, bacterial communities clustered separately (R2 = 0.361; Pr(> F) = 0.001), albeit heterogeneous dispersion (Pr(> F) = 0.004) was noticed (Fig. 4b). Tissues differed from DNA extraction negatives (R2 = 0.089; Pr(> F) = 0.001) and PCR negatives (R2 = 0.054; Pr(> F) = 0.020), but pairwise comparison with paraffin did not meet the condition of homogenous dispersion. No significant differences were found between paraffin and PCR negatives (R2 = 0.072; Pr(> F) = 0.219). Analyses were also performed at OTU level, which showed similar results (Supplementary Table S3). Multiple bacterial genera were shared between tissues, paraffin and PCR negatives, but not with DNA extraction negatives and mock controls (Fig. 4c). Six bacterial families were found in DNA extraction negatives (Supplementary Fig. S2a). To estimate the accuracy of the experimental procedure and the pipeline, Pearson correlation for theoretical and experimental mock communities was calculated. The correlation was r = 0.923 (*P =* 0.064) and r = 0.952 (*P =* 0.064) for the mock controls, respectively (Supplementary Fig. S2b).

### Retrieving biologically relevant information from FFPE samples remains challenging

**Fig. 5:**
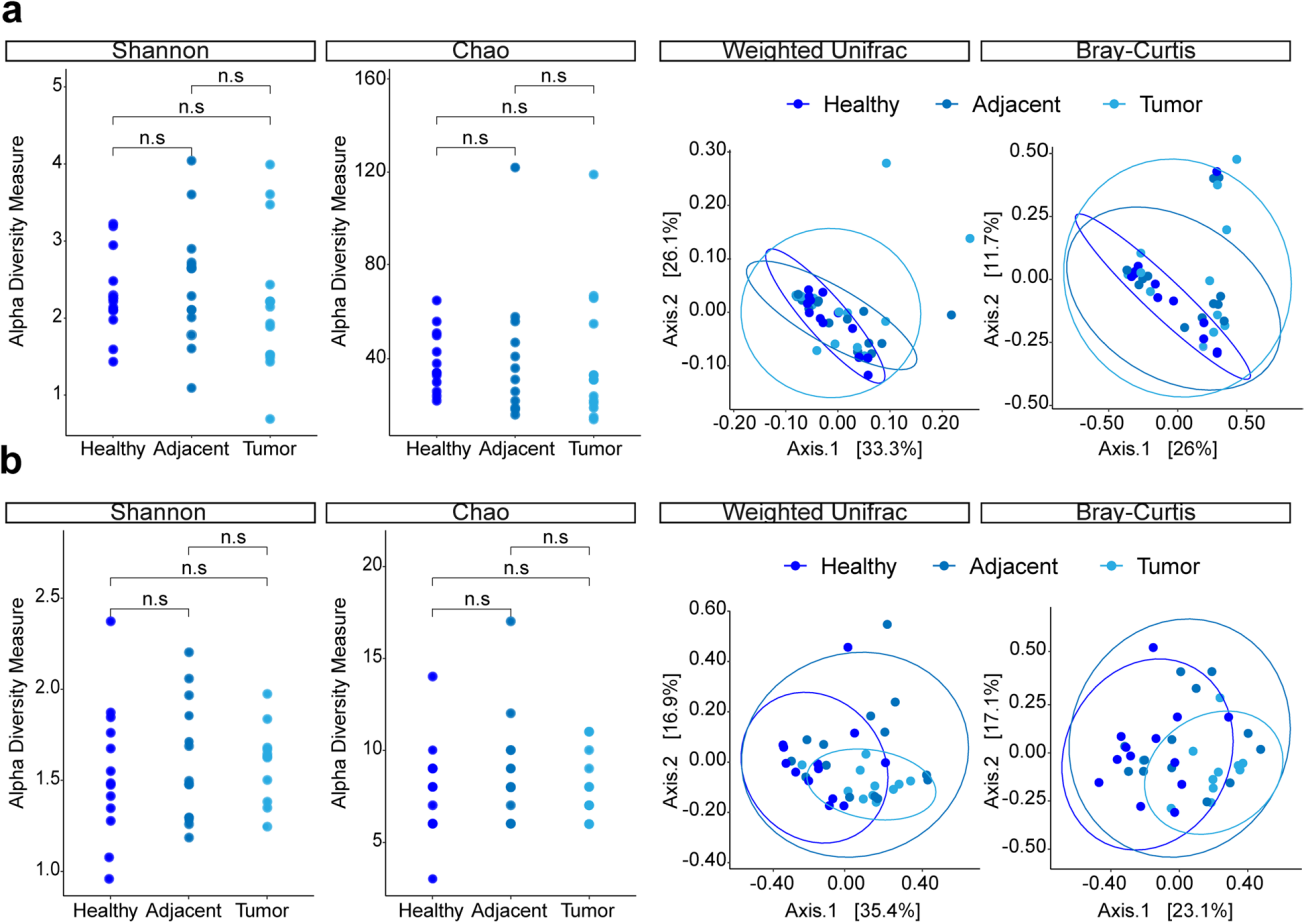
Bacterial diversity of tumor, normal adjacent and healthy control tissues following decontamination. **a-b** The α- and β- diversity results of formalin-fixed paraffin embedded (FFPE) tissues at genus level following decontamination are shown for the datasets obtained by a non-nested (**a**) and a nested (**b**) polymerase chain reaction (PCR) approach. The dot plots show the bacterial biodiversity measured by Shannon and Chao1 diversity indices. Principal Coordinates Analysis (PCoA) plots illustrate the bacterial composition of tissue using weighted UniFrac and Bray-Curtis dissimilarity. Abbreviations for level of significance: n.s. not significant

The library size of both datasets comprised of low read numbers overall for FFPE tissues which overlapped with DNA extraction negatives in the non-nested PCR data set and PCR negatives in the nested PCR dataset (Supplementary Fig. S3a,b). These controls were set for the identification of contaminants using the prevalence method at a threshold of 0.5 that was selected based on its discriminative ability (Supplementary Fig. S3c,d) [27]. This resulted in the removal of 1.006.017 (13.8 %) and 5.464.548 (22.4 %) reads corresponding to the biopsy samples in respectively the non-nested and nested PCR datasets. The remaining 1684 OTUs within the non-nested PCR dataset following decontamination belonged to 42 phyla including Proteobacteria (30.6 %), Firmicutes (29.2 %) and Bacteroidetes (14.4 %) while the nested PCR data set comprised of 440 OTUs from six phyla including Proteobacteria (45.2 %), Firmicutes (41.8 %) and Actinobacteria (8.2 %) in descending order (Supplementary Fig. S4a,b). Deinococcus-Thermus from both data sets and many spurious others from the non-nested PCR data set (e.g. Planctomycetes) are generally not seen in fecal samples [28], which might hamper the retrieval of biologically relevant information. Nevertheless, the detection of *Faecalibacterium* and *Escherichia-Shigella species* within tissue samples was indeed possible by sequencing. After decontamination, there were no differences in α- and β diversity between tumor, normal adjacent and healthy tissues in the non-nested dataset at genus level (Fig. 5a). The nested dataset failed to meet the homogeneity condition of permutational multivariate analyses of variance (PERMANOVA), thus rendering dispersion as the possible reason for significant differences in β-diversity (Fig. 5b).

## Discussion

FFPE tissue specimens may provide an important source to study the microbiota in health and disease, but their use is associated with several technical challenges. Our study demonstrates that despite the use of specialised bacterial DNA isolation kits, normalisation of low biomass FFPE tissue samples is primarily driven by human DNA rather than bacterial DNA presence. Our study also shows that the use of non-normalized (undiluted) template may improve bacterial detection in downstream bacterial analyses. Although bacterial marker analysis by qPCR was feasible for two selected markers (Fig. 2), high throughput analyses would require hundreds of individual qPCR assays and sequencing efforts may thus prove to be more efficient. Nevertheless, we show that the extraction of biological relevant information from 16S rRNA amplicon sequencing data remains difficult. Our sequencing pilot was distorted by a previously reported contaminant, *Ralstonia* [22, 25, 26, 29], underscoring the critical impact of bacterial contamination on low biomass samples as described by others [21–26]. We applied and recommend stringent measures for processing FFPE tissues to reduce the chance of contamination (Supplementary Fig. S5), but it should be noted that PCR negatives not undergoing DNA extraction also showed significant presence of bacterial DNA, which were either introduced during PCR and sequencing efforts, or the result of sequencing artefacts arising as a result of low biomass (Fig. 4c). In particular the detection of biologically relevant taxa such as the Bifidobacteriaceae, Lactobacillaceae and Enterobacteriaceae families in negative controls (Supplementary Fig. S2a) can reduce the robustness concerning the presence of such taxa in samples. Since negative controls demonstrate both the nature and the source of contamination, quality control at different steps of sequencing efforts is particularly recommended. The inclusion of paraffin controls allowed us to demonstrate that bacterial communities of tissues and paraffin tend to cluster separately at genus level in both datasets, and therefore paraffin seems less informative regarding to contaminants. However, the incorporation of DNA extraction negatives and PCR negatives showed that these low biomass samples (tissue and paraffin) were highly affected by bacterial contaminants and/or sequencing artefacts, in line with recent literature [24, 30]. Synthetic mock controls with high bacterial biomass were less affected, but future studies should consider mock controls with both high and low concentration [31]. Controls with similar microbial biomass as the experimental samples would be representative for the actual effect of contamination and the loss of specific signal due to sequencing efforts.

Knowledge of the characteristics of the investigated ecosystem and the introduction of controls is important for the critical appraisal of results [22]. The DNA quality obtained from FFPE tissue specimens allowed bacterial marker analysis by qPCR. In addition to the detection of *E.coli* and *ClbA* gene positive strains, the finding of lower *F. prausnitzii* levels in CRC compared to healthy controls is in accordance to previous findings [32, 33]. Our sequencing efforts indeed indicated the presence of *Escherichia* spp and *Faecalibaterium* spp in a part of the FFPE tissues. The finding of Proteobacteria as the major phylum in both datasets is not fully understood, but in line with a recent study suggesting that the paraffin embedding process might influence the microbial profile [34]. Multiple aspects in different processes involving pre-processing of tissues, storage conditions, DNA isolation, library preparation and actual sequencing are known to influence the outcome [21, 24–26]. While FFPE tissue specimens have previously been used for 16S rRNA amplicon sequencing [8, 10] and also for shotgun metagenomics study recently [35], data retrieval is limited compared to frozen tissues [34]. Nevertheless, FFPE samples are sometimes the only available source to answer research questions, allowing complementary taxonomic and functional exploration the microbiome despite relatively low read counts [35]. Thus, when using this material, we highly recommend the identification of prominent contaminating sources to increase the robustness of the dataset’s biological information.

Our study has several limitations. First, our bacterial amplification approaches were not directly comparable due to different library preparations and sequencing platforms. Although PCR negatives and mock controls were not available in both sequencing efforts, their findings together emphasized the importance to include both positive and negative controls to account for bacterial contamination. Secondly, the retrieval of biological relevant information from low abundance bacterial DNA of questionable quality and quantity remains challenging. The maximum DNA fragment size detectable was not determined for each of our DNA samples, but it might be helpful to guide the experimental set-up in future studies using FFPE tissues. Although the exploration of alternative tissue fixation and isolation protocols was also out of our scope, bacterial marker analysis by qPCR with FFPE tissue specimens was possible here. Thirdly, the decontamination process leads to loss of both data in general and possibly rare bacterial taxa in low bacterial biomass samples, which should be taken into consideration by researchers when extrapolating such results. Lastly, individual laboratory reagents for library preparation were not tested, but the inclusion PCR negatives are essential to account for procedural related contamination and to interpret results. The application of enzymatic treatment of PCR master mixes has been suggested [26], and might be considered in future efforts.

## Conclusions

Our study with FFPE tissue specimens has stressed the importance to implement measures against bacterial contamination in microbiome research with low bacterial biomass samples. Since human genomic DNA is being co-extracted from FFPE tissues, the use of non-normalized (undiluted) template is recommended for bacterial detection in downstream molecular analyses. While the execution of 16S rRNA amplicon sequencing on FFPE tissue specimens remains difficult, the inclusion of negative controls (e.g. DNA extraction negatives and PCR negatives) and positive controls (e.g. synthetic mock communities) is important for quality control, and the use of only one source of contamination control may not be sufficient. Future microbiome studies with low biomass specimens should critically evaluate laboratory procedures to account for bacterial contamination.

## Methods

### FFPE tissue and paraffin collections

FFPE colonic tissue specimens (n = 8) were used for our initial 16S rRNA amplicon sequencing pilot. A total of 41 FFPE tissue specimens, containing CRC (n = 14), paired normal adjacent tissue (n = 14) and healthy colonic tissue (n = 13), were collected for bacterial marker analysis and 16 S rRNA amplicon sequencing. Microscopic findings were confirmed by an expert gastrointestinal pathologist. All FFPE tissue blocks were processed in neutral-buffered formalin and embedded with paraffin during routine medical practice and obtained from the department of pathology at the Erasmus MC University Medical Center Rotterdam, the Netherlands. In addition, paraffin was sampled from six sources including two batches of paraffin grains, one tissue processor machine and three paraffin embedding stations and transferred into clean autoclaved bottles until bacterial DNA isolation.

### Microtome sectioning

A regular cleaning protocol was applied for processing samples of our 16S rRNA amplicon sequencing pilot, entailing the use of ethanol to clean the microtome and metal tweezers before sectioning. For each block, 14 consecutive sections of 5µM were obtained after disposal of the superficial layers and transferred in autoclaved 1.5 ml Eppendorf tubes for storage until bacterial DNA isolation. A second more stringent contamination-prevention protocol including DNA-Zap treatment of all surfaces and the use of facemasks and flow cabinets was employed to process following specimens (Supplementary methods 1). To control for potential contamination in downstream analysis, paired empty paraffin (0.05 gram) from the same FFPE tissue block was collected using a sterile disposable surgical knife and a clean weighing scale. All specimens were cut within several days of each other, placed in Eppendorf tubes and transferred into a dark box for storage in a cold room to prevent degradation by light/heat until DNA isolation within a few weeks.

### Bacterial DNA extraction

Bacterial DNA isolation of FFPE tissues and paraffin was carried out with the RTP Bacteria DNA Mini Kit (STRATEC Molecular Gm, Berlin, Germany) according to the manufacturer’s protocol for FFPE material. The first step was modified using xylene to dissolve paraffin. Melted paraffin (100 µl) from six pathology sources served as starting material for DNA isolation with two RTP Bacteria Mini Kits. Autoclaved water (500 µl) and/or blank (no template) samples that were processed by the RTP Bacteria DNA Mini Kit served as additional controls.

For bacterial marker analysis and 16S rRNA amplicon sequencing, FFPE tissue and their paired empty paraffin samples were concurrently processed for DNA extraction in a non-specific order using a stringent decontamination protocol (Supplementary methods 1). To prevent repetitive pipetting steps, each DNA sample was divided in aliquots of which one was utilized for 16S rRNA amplicon sequencing. DNA purity was measured with NanoDrop 2000 Spectrophotometer (Thermo Fisher Scientific Inc., Waltham, MA) and concentration with Qubit dsDNA BR Assay Kit (Thermo Fisher). Samples were stored at -20 °C until further analysis.

### Polymerase chain reaction

All samples and potential contamination sources, e.g. individual components of three individual RTP Bacteria DNA Mini Kits, extraction additives xylene, ethanol and isopropanol and paraffin were subjected to PCR amplification. PCR assays were executed with an Applied Biosystems 2720 Thermal Cycler (Applied Biosystems, Waltham, MA) using primers targeting the *16S rRNA* gene, the human *beta*-*actin (ACTB)* gene and *Ralstonia* species (Supplementary Table S1). Each PCR reaction contained GoTaq® buffer (Promega, Madison, WI), 1.25mM MgCl_2_ (Promega), 0.167mM (each) deoxynucleotides (Roche Diagnostics, Mannheim, Germany), 2.5U GoTaq®polymerase (Promega), 333nM of each primer (Sigma-Aldrich, St Louis, MO), 2 µl of template and water to a final volume of 30 µl. After 4 min of denaturation at 95 °C, 40 cycles consisting of 30 s denaturation at 95 °C, 30 s annealing and 1 min extension at 72 °C were applied, and followed by the final extension of 10 min at 72 °C. Template was not normalized (undiluted) or normalized to 10ng/µl where otherwise specified. Water served as negative PCR control and positive controls were fecal bacterial DNA, human genomic DNA from FFPE tissues and known *Ralstonia-*contaminated elution buffer. Amplicons were visualized by gel electrophoresis using 2 % agarose gel in 1X TBE (Tris-borate-EDTA) buffer containing Serva DNA stain G (Promega).

### Quantitative polymerase chain reaction assays

Primer details for qPCR assays are described in Supplementary Table S1. To determine bacterial versus human genomic DNA concentration within FFPE tissue samples (n = 3), a standard curve with equimolar *Escherichia coli* (*E.coli)* and human genomic DNA was prepared (Supplementary methods 2). The reaction mixture comprised of SYBR Select Master Mix (Applied Biosystems), 200-500nM of each primer (Sigma-Aldrich), 2 µl of normalized template (10ng/µl) and water for a total volume of 20 µl and DNA was amplified using the same cycles as described above. The bacterial versus human genomic DNA concentration were calculated using their respective standard curves and illustrated with Graph Pad Prism 5 software (GraphPad, San Diego, CA). Additionally, 16S rRNA gene copy numbers in paired tissue (n = 39) and empty paraffin (n = 38) samples were calculated and groups analysed with the Wilcoxon test.

Bacterial marker analysis was performed with *E.coli* and *ClbA* gene primers to detect *E.coli* and CRC associated genotoxic strains carrying the pathogenicity island *pks* [36, 37], respectively. Gut commensal *Faecalibacterium prausnitzii (F.prausnitzii)*, which has been reported to be negatively associated with CRC [32, 33], was additionally selected. To account for different FFPE tissue sizes, the *ACTB* gene was measured. PCR conditions were similar as described above, except for the use of 4 µl non-normalized template to enhance amplification. The 2^−ΔΔCT^ method was applied to calculate the fold change. The ΔCT_sample_ (= CT_bacterial target_ – CT_ACTB target_) was first obtained for each sample by normalization to the amount of total human DNA. The average ΔCT_sample_ of the healthy tissues (control group) was then used to calculate ΔΔCT (= ΔCT_sample_ – average ΔCT_control group_), after which the fold change derived from 2^−ΔΔCT^. The Kruskal-Wallis with the Dunn’s Multiple Comparison test for post-hoc analysis were performed in Graph Pad Prism 5.

### Library preparation and 16S rRNA amplicon sequencing

16S rRNA amplicon sequencing was performed at the Macrogen Institute, Seoul, Korea, using amplification of the 16 S rRNA hypervariable region V3-V4 by 341F/805R primers. Libraries included paired empty paraffin controls (n = 6) and DNA extraction negatives (n = 6) for sequencing on the Illumina MiSeq platform (2 × 300 bp) (Illumina, San Diego, CA). Secondly, a nested PCR approach was applied in house using 27 F/1369R and 515 F/806R primers for respectively the initial and a subsequent PCR targeting the V4 region. Paired empty paraffin (n = 41), synthetic bacterial mock communities (n = 2) and PCR negative controls (= 2) were concurrently processed with the tissues. DNA extraction negatives from the aforementioned RTP Bacteria Mini Kits (n = 6) were additionally prepared to allow in depth comparison of these controls with the non-nested data set. Sequencing was conducted on the Illumina NovaSeq 6000 platform (2 × 150 bp) at GATC Biotech (Konstanz, Germany). More details about library preparation and primer sequences are described in Supplementary methods 3 and Supplementary Table S2.

### 16S rRNA amplicon sequencing data processing

For both the 16S rRNA amplicon sequencing pilot and the two larger data sets (non-nested and nested PCR approaches), quality control of the reads was performed with FASTQC [38] in Java Runtime Environment and Rqc package [39] in R version 3.5.0 [40]. The NG-Tax pipeline with default settings was applied [41, 42]. The operational taxonomic unit (OTU) table was constructed at 0.1 % abundance threshold, unassigned reads with one mismatch included and chimeras removed. Taxonomic assignment was conducted with the USEARCH algorithm [43] against the Silva SSU 128 database [44]. Further analysis was performed in R with the ‘phyloseq’ [45], ‘microbiome’ [46] and ‘vegan’ [47] packages. Group comparison was conducted based on sample type (tissue, paraffin, controls). Alpha-diversity was computed with Shannon and Chao1 Indices while β-diversity was assessed with Principal Coordinates Analysis (PCoA) based on weighted UniFrac and Bray-Curtis dissimilarity at genus and OTU level after relative abundance transformation of the data. The ‘adonis’ permutational multivariate analyses of variance (PERMANOVA) was applied to determine statistical significance between groups. The ‘betadisper’ function was used to test for multivariate homogeneity of groups dispersons [48]. To ensure reproducibility, the seed was set to 995 for both permutations tests. The biological significance of the data sets was reassessed following removal of contaminants identified by the prevalence method of the ‘decontam’ package [27]. A 0.5 threshold was set and negative controls consisted of DNA extraction negatives for the non-nested PCR dataset and PCR negatives for the nested PCR approach.

## Supplementary Information

**Additional file 1.**

## Data Availability

16S rRNA amplicon sequences have been deposited in the NCBI SRA database under the BioProject ID PRJNA741803 and PRJNA742764.

## Abbreviations

ACTB: beta-actin
CRC: colorectal cancer
DNA: deoxyribonucleic acid
FFPE: formalin-fixed paraffin embedded
OTU: operational taxonomic unit
PCoA: Principal Coordinates Analysis
PCR: polymerase chain reaction
PERMANOVA: permutational multivariate analyses of variance
qPCR: quantitative polymerase chain reaction
16S rRNA: 16S ribosomal RNA
