# Supplementary material for: Technical challenges regarding the use of formalin-fixed paraffin embedded (FFPE) tissue specimens for the detection of bacterial alterations in colorectal cancer
Source: BMC Microbiol. 2021 Oct 29;21:297. doi: 10.1186/s12866-021-02359-z (PMC8555202; doi:10.1186/s12866-021-02359-z)
Supplement: Supplementary file 1 — Additional file 1. [file 12866_2021_2359_MOESM1_ESM.pdf]

Additional file - Supplementary information

Technical challenges regarding the use of formalin-fixed paraffin embedded (FFPE) tissue specimens for the detection of bacterial alterations in colorectal cancer

Supplementary methods

1. Protocol to process formalin-fixed paraffin embedded (FFPE) tissue specimens for bacterial analysis

Microtome sectioning of FFPE tissue specimens entailed the use of disposable surgical gowns, gloves and face masks. Prior to and between sectioning of each sample, the microtome was cleaned with ethanol and DNA *Zap*™ PCR DNA Degradation Solutions (Thermo Fisher Scientific Inc., Waltham, MA), rinsed with autoclaved demineralized water and wiped dry with surgical gauze. Blades and gloves were consistently changed between samples to prevent cross-contamination and sterile disposable surgical tweezers were used to place serial sections into autoclaved 1.5 ml Eppendorf tubes. Bacterial DNA isolation was carried out in a laminar flow cabinet to minimize environmental and aerogenic contamination, except for the first step which required a fume hood for xylene. All pipetting work was performed using Filter Tips after dissembling and thorough cleaning of the pipettes. Similar to other laboratory equipment, treatment with ethanol and DNA *Zap* solutions was followed by ultraviolet (UV) radiation in the laminar flow cabinet. Disposable surgical gowns and face masks were used and gloves were regularly changed when handling samples.

2. Standard curve for quantitative polymerase chain reaction (qPCR) analyses

A kanamycin-resistance gene containing *Escherichia coli* (*E.coli*) strain (DH5α) was cultured at 37°C in Luria Broth (LB) medium containing Kanamycin (50µg/ml) and DNA was isolated according to the protocol of the RTP Bacteria DNA Mini Kit (STRATEC Molecular Gm, Berlin, Germany) for bacterial cell pellets. Human DNA was extracted from a gastric epithelial cell (GES) line using the Wizard DNA Purification kit (Promega, Madison, WI). Both *E.coli* and human samples were subjected to the StepOnePlus Real-Time PCR system (Applied Biosystems, Waltham, MA) for PCR amplification with subsequent gel electrophoresis. The *E.coli* and human amplification products were purified from the agarose gel with the Monarch DNA Gel Extraction Kit (New England Biolabs Inc., MA), normalized to a concentration of 10ng/µl and combined in equivalent volumes to create a subsequent five 1:10 serial dilution with autoclaved water.

3. Library preparation and 16S ribosomal RNA (rRNA) amplicon sequencing

The V3-V4 region of the 16S rRNA gene was amplified at the Macrogen Institute using 341F-805R primers (**Supplementary Table S2**), which were attached to Illumina overhang adapters (forward: 5'-TCGTCGGCAGCGT CAGATGTGTATAAGAGACAG-3'; reverse 5'-GTCTCGTGGGCTCGGAGATGTGTATAAGAGACAG-3'). The reaction mixture consisted of 12.5µl 2x KAPA HiFi HotStart Ready Mix (Kapa Biosystems, Cape Town, South Africa), 200nM of each primer and 2.5µl of non-normalized template for a total volume of 25µl. After 3 minutes of initial denaturation at 95°C, 25 cycles of 30 seconds denaturation at 95°C, 30 seconds of extension at 55°C and 30 seconds of elongation at 72°C took place, and ended with 5 minutes of final extension at 72°C. Following PCR clean-up with AMPure XP beads (Agencourt Bioscience Corporation, Beverly, MA), multiplexing indices and Illumina sequencing adapters were attached in an index PCR using the Nextera XT Index Kit (Illumina, San Diego, CA). After 8 cycles under the above described PCR conditions, the reaction mixture was purified with the AMPure XP beads. The final library was normalized and sequenced on the MiSeq system (Illumina) to produce 2x300bp end reads.

A nested PCR approach was conducted in house using consecutive 27F/1369R and 515F/806R primer pairs (**Supplementary Table S2**). The reaction mixture of the first PCR contained 10µl 5x Phusion HF buffer (ThermoFisher Scientific, Waltham, MA), 0.5µl Phusion Hot Start II DNA polymerase (ThermofFisher Scientific), 200µM deoxynucleotides, 100nM of each primer, 1µl non-normalized template and 36.5µl water for a total volume of 50µl. After 30 seconds of initial denaturation at 98°C, 25 cycles of 10 seconds denaturation at 98°C, 10 seconds annealing at 50°C and 30 seconds extension at 72°C were applied, and followed by 10 minutes of final extension at 72°C. After amplicon purification with the DNA Clean & Concentrator Kit (Zymo Research, Irvine, CA), 1µl template was subjected to a second PCR targeting the V4 region of the 16S rRNA gene using primers which were tagged with a barcode at the 5'end for recognition. PCR amplification was performed in triplicate under the following conditions: 30 seconds of denaturation at 98°C, 25 cycles of 10 seconds denaturation at 98°C, 10 seconds annealing at 50°C and 10 seconds of extension at 72°C, and final extension of 10 minutes at 72°C. Amplicons were visualized on 1% agarose gel for verification, pooled and purified using the CleanPCR kit (CleanNA, Waddinxveen, the Netherlands). Only amplicons of FFPE tissue and paraffin controls samples that were verified on agarose gel were included for sequencing on the Illumina NovaSeq 6000 platform (Illumina) to produce 2x150bp paired-end reads at GATC Biotech (Konstanz, Germany).

Supplementary Table S1 Overview of primers used for (quantitative) polymerase chain reaction analysis

**Target**	**Sequence**	**T_a** (°C)	**Size** (bp)	**Ref**
Ralstonia species[a]	F: 5'-CTGGGGTCGATGACGGTA-3'<br>R: 5'-ATCTCTGCTTCGTTAGTGGC-3'	56	546	**1**
ACTB gene[a,b,c]	F: 5'-CTGGAACGGTGAAGGTGACA-3'<br>R: 5'-AAGGGACTTCCTGTAACAATGCA-3'	60.5	140	**2**
16S rRNA gene[a,b]	F: 5'-CGGTGAATACGTTCCCGG-3'<br>R: 5'-TACGGCTACCTTGTTACGACTT-3'	60	145	**3-4**
16S rRNA gene[d]	F: 5'-GTGSTGCAYGGYYGTCGTCA-3'<br>R: 5'-ACGTCRTCCMCNCCTTCCTC-3'	52	147	**5**
E.coli[c]	F: 5'-CATGCCGCGTGTATGAAGAA-3'<br>R: 5'-CGGGTAACGTCAATGAGCAAA-3'	62	96	**6**
E.coli (ClbA gene)[c]	F: 5'-ATGAGGATTGATATATTAATTGGACA-3'<br>R: 5'-GGTTTGCCATATTTGCACGTAC-3'	58	233	**7-8**
F. prausnitzii[c]	F: 5'-GATGGCCTCGCGTCCGATTAG-3'<br>R: 5'-CCGAAGACCTTCTTCCTCC-3'	58	198	**9**

[a] End-point PCR [b] qPCR to determine human versus bacterial DNA concentrations [c] qPCR for bacterial marker analysis [d] qPCR to determine 16S rRNA copy numbers

Abbreviations: ACTB, *beta-actin*; bp, base pair; *E.coli, Escherichia coli; F.prausnitzii, Faecalibacterium prausnitzii*; F, forward; R, reverse; Ref, reference; T_a, annealing temperature.

References

1. Coenye T, Spilker T, Reik R, Vandamme P, Lipuma JJ: Use of PCR analyses to define the distribution of Ralstonia species recovered from patients with cystic fibrosis. *J Clin Microbiol* 2005, 43(7):3463-3466.
2. Vandesompele J, De Preter K, Pattyn F, Poppe B, Van Roy N, De Paepe A, Speleman F: Accurate normalization of real-time quantitative RT-PCR data by geometric averaging of multiple internal control genes. *Genome Biol* 2002, 3(7):RESEARCH0034.
3. Suzuki MT, Taylor LT, DeLong EF: Quantitative analysis of small-subunit rRNA genes in mixed microbial populations via 5'-nuclease assays. *Appl Environ Microbiol* 2000, 66(11):4605-4614.
4. Furet JP, Firmesse O, Gourmelon M, Bridonneau C, Tap J, Mondot S, Dore J, Corthier G: Comparative assessment of human and farm animal faecal microbiota using real-time quantitative PCR. *FEMS Microbiol Ecol* 2009, 68(3):351-362.
5. Maeda H, Fujimoto C, Haruki Y, Maeda T, Kokeguchi S, Petelin M, Arai H, Tanimoto I, Nishimura F, Takashiba S: Quantitative real-time PCR using TaqMan and SYBR Green for Actinobacillus actinomycetemcomitans, Porphyromonas gingivalis, Prevotella intermedia, tetQ gene and total bacteria. *FEMS Immunol Med Microbiol* 2003, 39(1):81-86.
6. Huijsdens XW, Linskens RK, Mak M, Meuwissen SG, Vandenbroucke-Grauls CM, Savelkoul PH: Quantification of bacteria adherent to gastrointestinal mucosa by real-time PCR. *J Clin Microbiol* 2002, 40(12):4423-4427.
7. Prorok-Hamon M, Friswell MK, Alswied A, Roberts CL, Song F, Flanagan PK, Knight P, Codling C, Marchesi JR, Winstanley C *et al*: Colonic mucosa-associated diffusely adherent afaC+ Escherichia coli expressing lpfA and pks are increased in inflammatory bowel disease and colon cancer. *Gut* 2014, 63(5):761-770.
8. Eklöf V, Löfgren-Burström A, Zingmark C, Edin S, Larsson P, Karling P, Alexeyev O, Rutegård J, Wikberg ML, Palmqvist R: Cancer-associated fecal microbial markers in colorectal cancer detection. *Int J Cancer* 2017, 141(12):2528-2536.
9. Ahmed S, Macfarlane GT, Fite A, McBain AJ, Gilbert P, Macfarlane S: Mucosa-associated bacterial diversity in relation to human terminal ileum and colonic biopsy samples. *Appl Environ Microbiol* 2007, 73(22):7435-7442.

Supplementary Table S2 Overview of primer sequences to prepare 16S ribosomal RNA amplicon sequencing libraries

**Target**	**Sequence**	**Ref**
341F-805R[a]	F: 5'-CCTACGGGNGGCWGCAG-3'<br>R: 5'-GACTACHVGGGTATCTAATCC-3'	**1 - 2**
27F-1369R[b]	F: 5'-AGAGTTTGATCMTGGCTCAG-3'<br>R: 5'-GCCCGGGAACGTATTCACCG-3'	**3 - 5**
515F-806R[b]	F: 5'-GTGYCAGCMGCCGCGGTAA-3'<br>R: 5'-GGACTACNVGGGTWTCTAAT-3'	**6 - 8**

[a] Primers used at the Macrogen Institute [b] Primers used in house

Abbreviations: F, forward; R, reverse; Ref, reference.

References

1. Herlemann DP, Labrenz M, Jurgens K, Bertilsson S, Waniek JJ, Andersson AF: Transitions in bacterial communities along the 2000 km salinity gradient of the Baltic Sea. *Isme J* 2011, 5(10):1571-1579.
2. Klindworth A, Pruesse E, Schweer T, Peplies J, Quast C, Horn M, Glöckner FO: Evaluation of general 16S ribosomal RNA gene PCR primers for classical and next-generation sequencing-based diversity studies. *Nucleic Acids Research* 2012, 41(1):e1-e1.
3. Weisburg WG, Barns SM, Pelletier DA, Lane DJ: 16S ribosomal DNA amplification for phylogenetic study. *J Bacteriol* 1991, 173(2):697-703.
4. Iwamoto T, Tani K, Nakamura K, Suzuki Y, Kitagawa M, Eguchi M, Nasu M: Monitoring impact of in situ biostimulation treatment on groundwater bacterial community by DGGE. *FEMS Microbiol Ecol* 2000, 32(2):129-141.
5. Yu Z, Morrison M: Comparisons of different hypervariable regions of rrs genes for use in fingerprinting of microbial communities by PCR-denaturing gradient gel electrophoresis. *Appl Environ Microbiol* 2004, 70(8):4800-4806.
6. Caporaso JG, Lauber CL, Walters WA, Berg-Lyons D, Lozupone CA, Turnbaugh PJ, Fierer N, Knight R: Global patterns of 16S rRNA diversity at a depth of millions of sequences per sample. *Proc Natl Acad Sci U S A* 2011, 108 Suppl 1:4516-4522.
7. Apprill A, McNally S, Parsons R, Weber L: Minor revision to V4 region SSU rRNA 806R gene primer greatly increases detection of SAR11 bacterioplankton. *Aquat Microb Ecol* 2015, 75:129-137.
8 Parada, AE., Needham, DM., Fuhrman, JA: Every base matters: assessing small subunit rRNA primers for marine microbiomes with mock communities, time series and global field samples. *Environmental Microbiology* 2016, 18(5), 1403–1414.

Supplementary Table S3. Comparison of bacterial communities from different groups within non-nested and nested polymerase chain reaction (PCR) data sets on operational taxonomic unit (OTU) level

**Non nested PCR dataset**		**Weighted UniFrac**	**Bray Curtis**
Overall	Pr(>F)	0.011	0.001
	R^2	0.086	0.109
	Homogeneity Pr(>F)	0.723	0.852
Tissues vs. paraffin	Pr(>F)	0.055	0.001
	R^2	0.045	0.062
	Homogeneity Pr(>F)	0.46	0.702
Tissues vs. DNA extraction negatives	Pr(>F)	0.019	0.001
	R^2	0.060	0.076
	Homogeneity Pr(>F)	0.733	0.737
Paraffin vs. DNA extraction negatives	Pr(>F)	0.565	0.319
	R^2	0.076	0.097
	Homogeneity Pr(>F)	0.644	0.303
**Nested PCR dataset**		**Weighted UniFrac**	**Bray Curtis**
Overall	Pr(>F)	0.001	0.001
	R^2	0.374	0.345
	Homogeneity Pr(>F)	0.155	0.003
Tissues vs. paraffin	Pr(>F)	0.001	0.001
	R^2	0.299	0.289
	Homogeneity Pr(>F)	0.067	0.001
Tissues vs. PCR negatives	Pr(>F)	0.058	0.030
	R^2	0.055	0.049
	Homogeneity Pr(>F)	0.931	0.465
Tissue vs. DNA extraction negatives	Pr(>F)	0.001	0.001
	R^2	0.155	0.086
	Homogeneity Pr(>F)	0.482	0.394
Tissues vs. positive control	Pr(>F)	0.002	0.002
	R^2	0.103	0.101
	Homogeneity Pr(>F)	0.024	0.007
Paraffin vs. PCR negatives	Pr(>F)	0.234	0.208
	R^2	0.061	0.072
	Homogeneity Pr(>F)	0.701	0.354

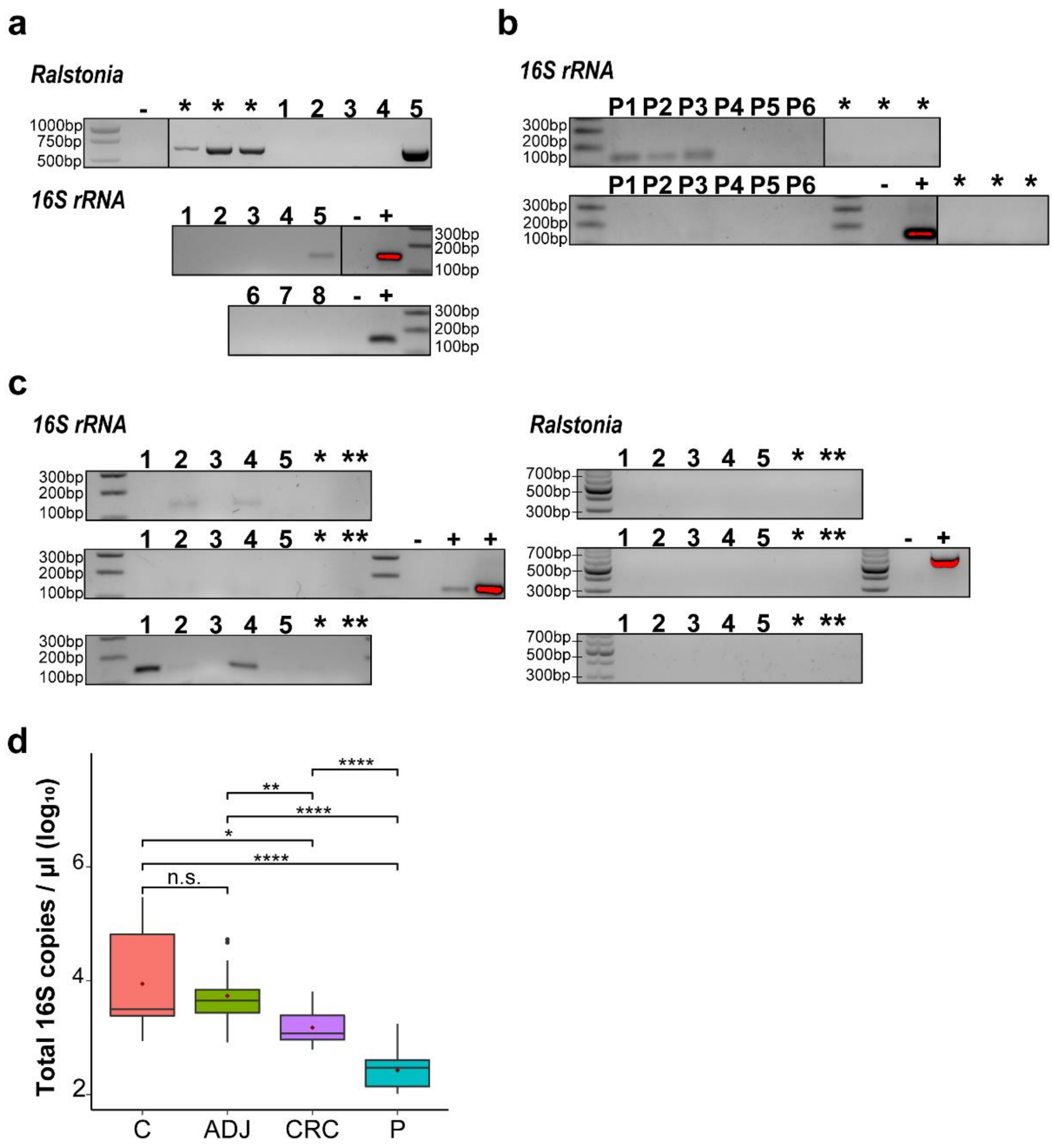

Supplementary Fig. S1 Bacterial analysis to examine extraction reagents and paraffin collections and to determine 16S rRNA gene copies in FFPE tissues. (a-c) Gel electrophoresis results following amplification with *Ralstonia* and/or *16S rRNA* gene primers to investigate bacterial presence in resuspension (1), binding (2), wash I-II (3-4) and elution (5) buffers of the RTP Bacteria DNA Mini Kit, extraction additives xylene (6), ethanol (7) and isopropanol (8), in addition to a set of paraffin collections (grains [P1-2], tissue processor machine [P3], embedding stations [P4-6]) processed by different kits. The individual gels in sections b and c represent the results obtained with two (b; upper and lower) and three (c; upper, middle and lower panel) different DNA isolation kits, respectively. The water (*) and blank (**) controls extracted with the DNA isolation kit were included, as well as positive (+) and negative (-) controls. **(d)** Quantitative polymerase chain reaction (qPCR) findings showing total *16S rRNA* gene copy numbers per µl DNA ($\log_{10}$) in healthy tissue controls (C; n=12), normal adjacent tissue (ADJ; n=14), colorectal cancer (CRC; n=13) and paraffin controls (P; n=38). The mean value is shown for each group. Abbreviations for level of significance: n.s. not significant; *, $P<0.05$; **, $P<0.01$; ****, $P<0.0001$. Full length gel electrophoresis results are shown in Additional Fig. A2

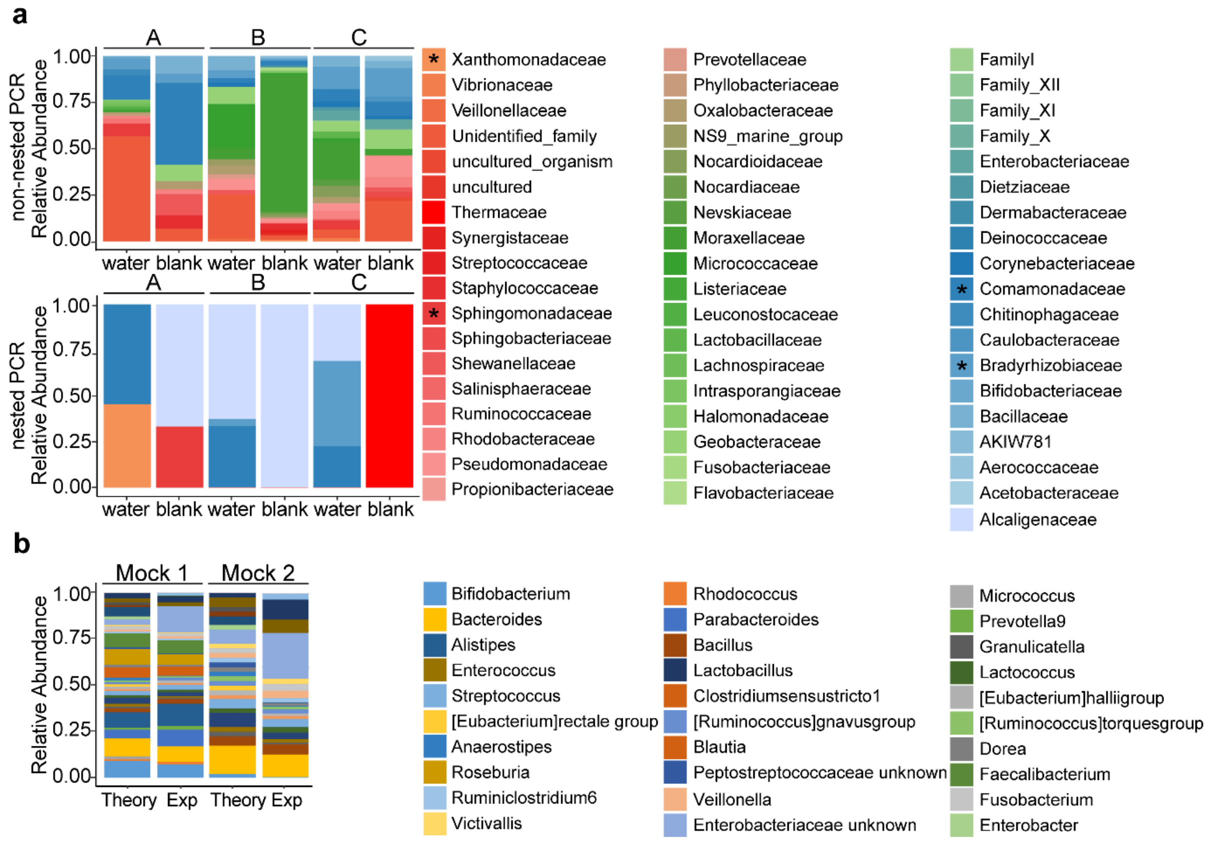

Supplementary Fig. S2 The relative abundance of bacterial families and genera in respectively DNA extraction negatives and mock controls. **(a)** The relative abundance plots at family level of DNA extraction negatives (n=6) that were processed with the different RTP Bacteria DNA Mini Kits A, B and C. The results of two different 16S rRNA amplicon sequencing approaches are shown. The non-nested PCR dataset demonstrated the presence of 53 bacterial families within the controls while a total of 6 families were detected in the nested PCR dataset. The asterisks (*) indicates bacterial families that were detected by both methods. **(b)** The relative abundance plot of theoretical (Theory) and experimental (Exp) mock controls of the nested dataset at genus level. The experimental mock communities underwent library preparation and 16S rRNA amplicon sequencing.

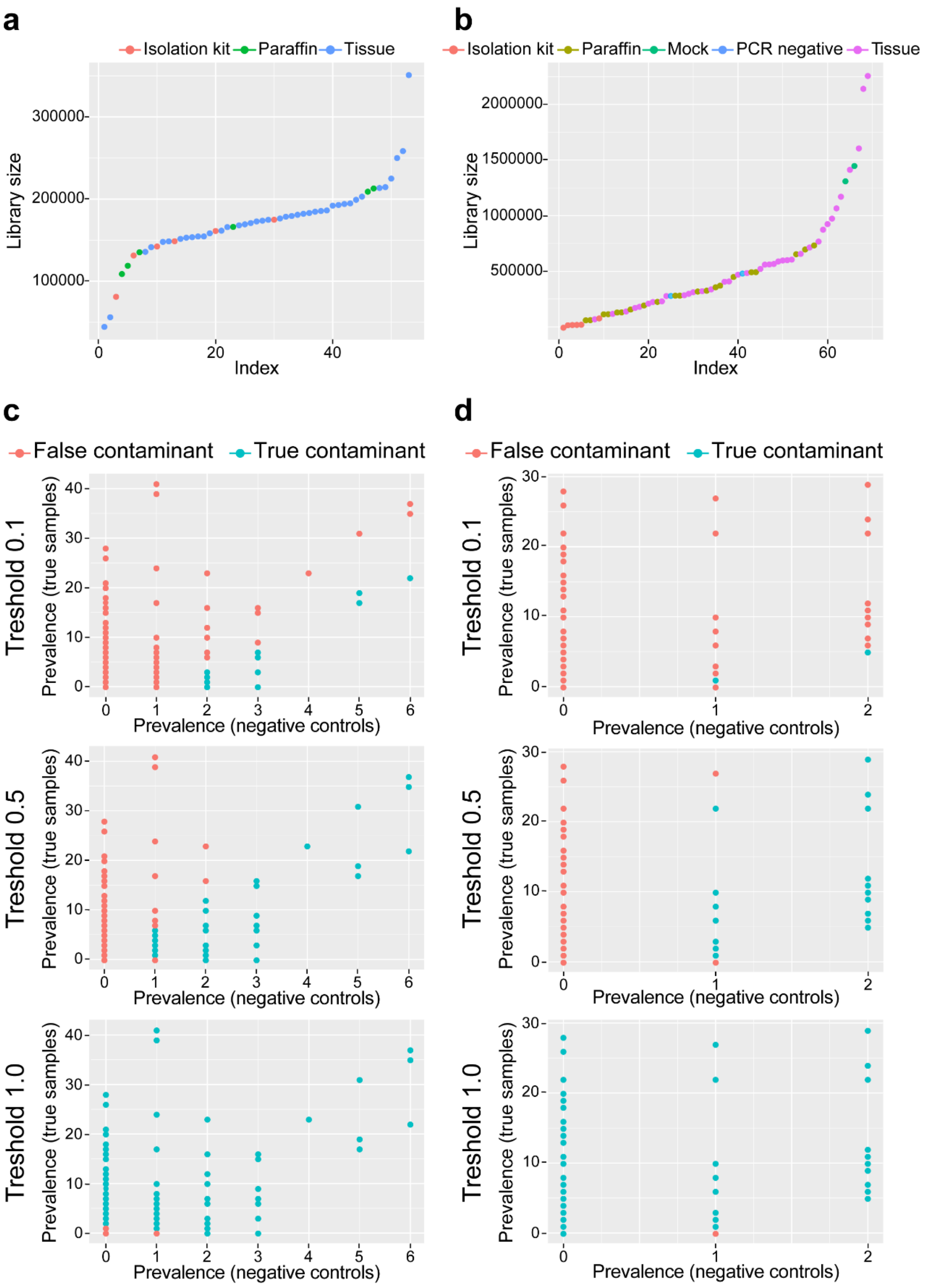

Supplementary Fig. S3 Library size and decontamination thresholds for the 16S rRNA amplicon sequencing data sets conducted with non-nested and nested polymerase chain reaction (PCR) approaches (a,b) The library size of samples from the non-nested (a) and nested (b) PCR data sets **(c,d)** The plots represents the decontamination treshold 0.1 (default) and the more stringent thresholds 0.5 and 1.0 for the identification of contaminants by the prevalence method. The negative controls comprised of DNA extraction negatives in the non-nested data set (c) and PCR negatives in the nested data set (d).

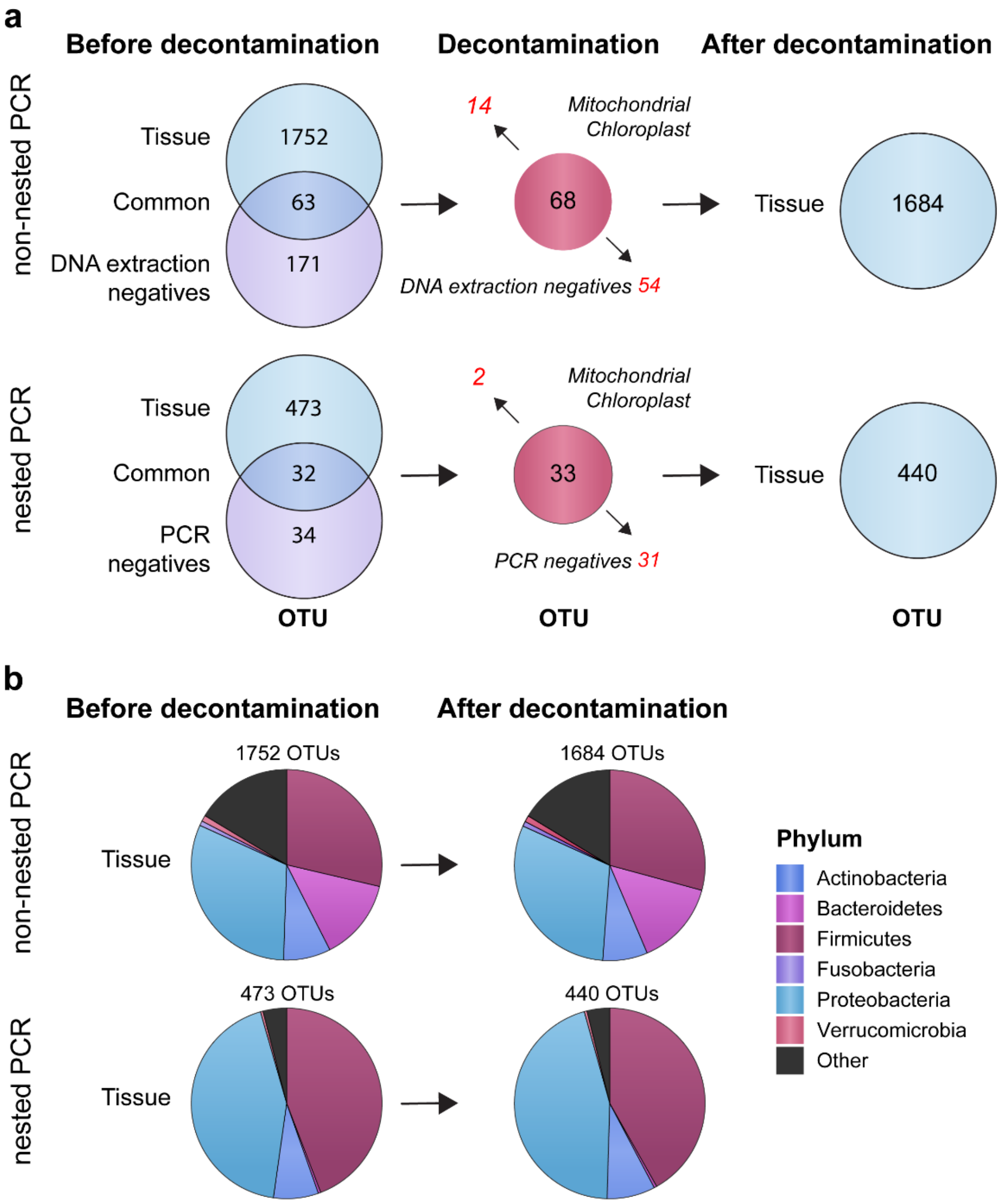

Supplementary Fig. S4 Decontamination process of the 16S rRNA amplicon data sets conducted with the non-nested and nested PCR approach (a) The number of operational taxonomic units (OTUs) within tissues are shown before and after decontamination. The DNA extraction negatives in the non-nested data set and PCR negatives in the nested data set were used for decontamination based on the prevalence method. **(b)** The plot shows the phyla to which the different OTUs belong.

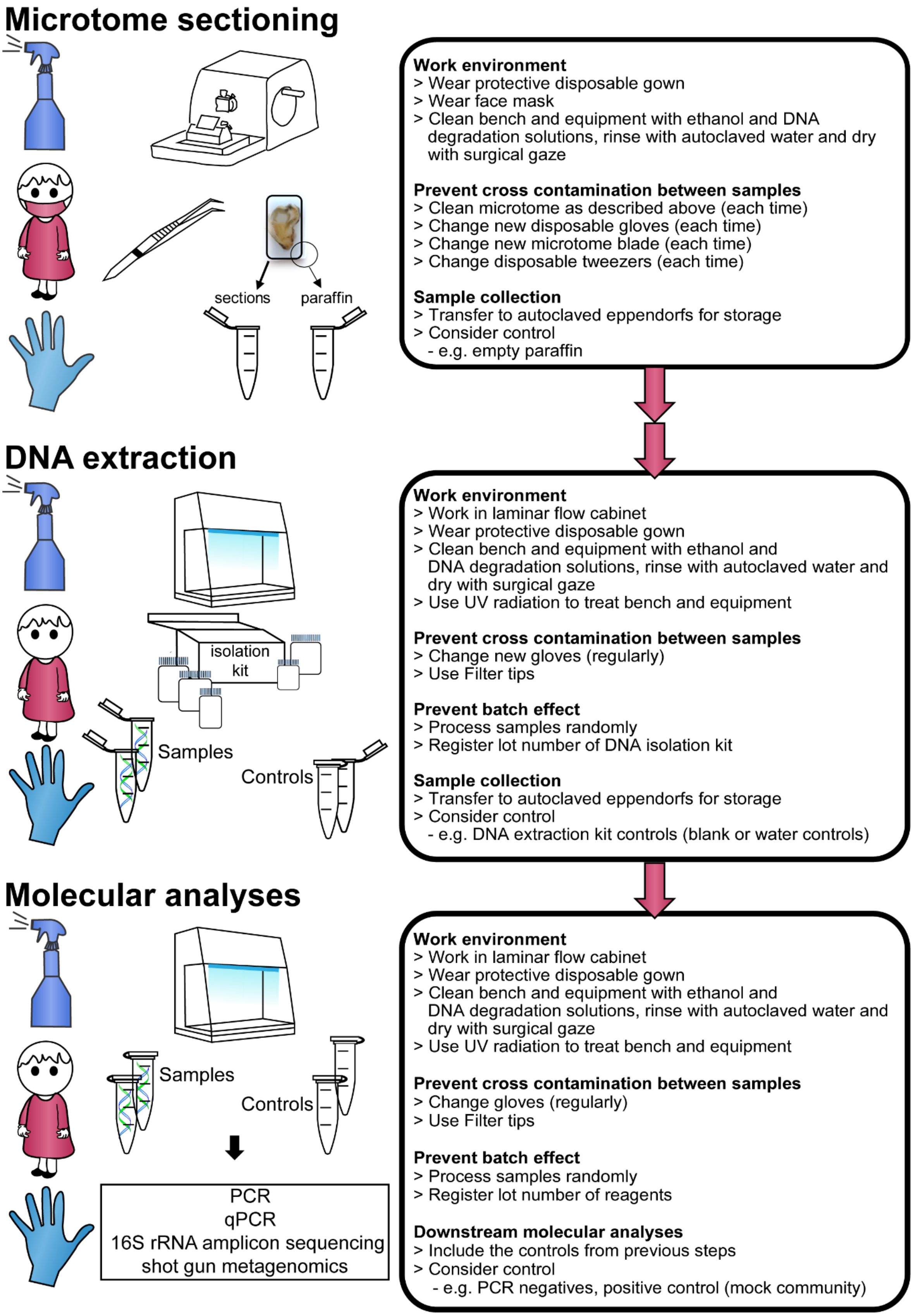

Supplementary Fig. S5 Processing formalin-fixed paraffin embedded (FFPE) tissue samples for microbial analyses. An overview of recommendations for handling FFPE tissue samples of low microbial biomass for downstream molecular analyses.

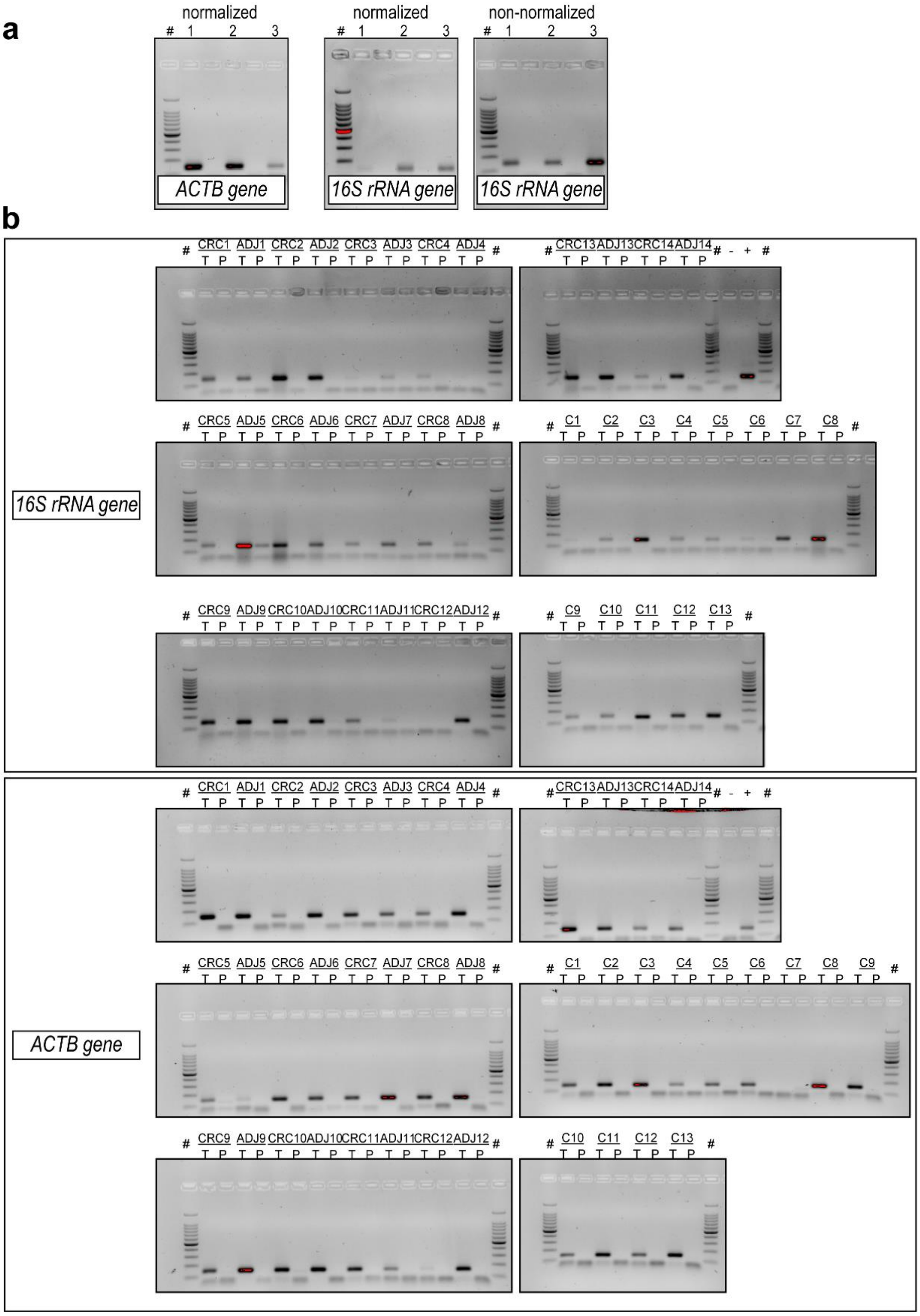

Additional Fig. A1 Full length gel electrophoresis results. (a) Gel results following *actin beta (ACTB)* and *16S rRNA* gene amplification, emphasizing the use of non-normalized (undiluted) template to improve bacterial DNA detection in FFPE tissue samples (n=3). **(b)** Results showing bacterial and human genomic DNA presence in 41 paired FFPE tissue (T) samples and their paired empty paraffin (P) controls. Tissues consist of colorectal cancer (CRC; n=14), normal adjacent (ADJ; n=14) and healthy control (C; n=13) tissues. The hash tack (#) represents the 100bp ladder. These uncropped findings are complementary to Fig. 1c-e.

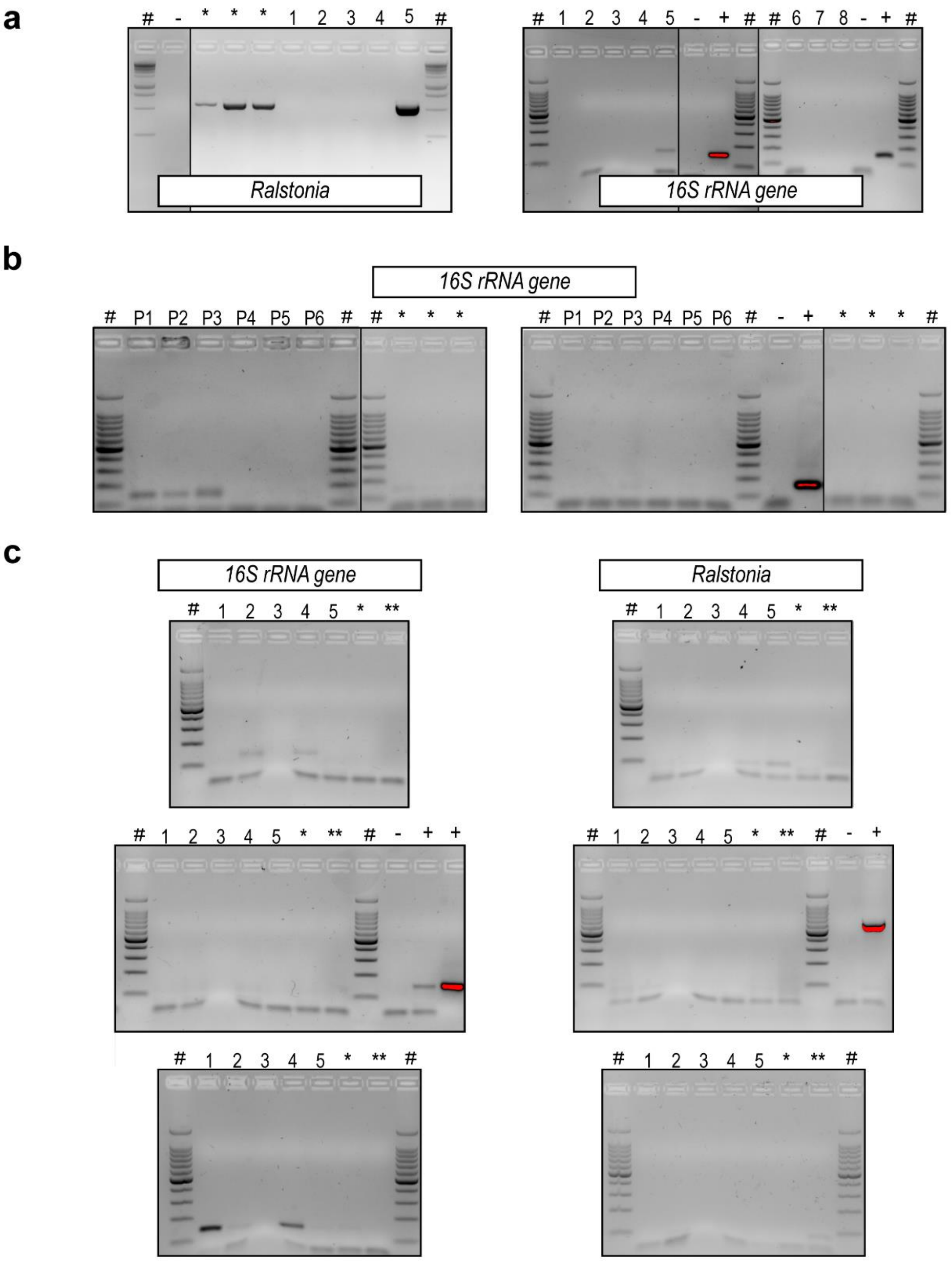

Additional Fig. A2 Full length gel electrophoresis results. (a-c) Gel results following amplification with *Ralstonia* and/or *16S rRNA* gene primers to investigate bacterial presence in resuspension (1), binding (2), wash I-II (3-4) and elution (5) buffers of the RTP Bacteria DNA Mini Kit, extraction additives xylene (6), ethanol (7) and isopropanol (8), in addition to a set of paraffin collections (grains [P1-2], tissue processor machine [P3], embedding stations [P4-6]) processed by different kits. The results in sections b and c represent the findings obtained with two (b; left and right pane) and three (c; upper, middle and lower panel) different DNA isolation kits, respectively. The water (*) and blank (**) controls extracted with the DNA isolation kit were included, as well as positive (+) and negative (-) controls. The hash tack (#) represents a

1kb ladder (a; left panel) or 100bp ladder (a [right panel], b, c). These uncropped findings are complementary to Supplementary Fig. S1a-c.
